# Genetic Variation in the Bitter Receptors Responsible for Epicatechin Detection Are Associated with BMI in an Elderly Cohort

**DOI:** 10.3390/nu13020571

**Published:** 2021-02-09

**Authors:** Alexandria Turner, Martin Veysey, Simon Keely, Christopher J. Scarlett, Mark Lucock, Emma L. Beckett

**Affiliations:** 1 School of Environmental and Life Sciences, University of Newcastle, Ourimbah 2258, Australia; c.scarlett@newcastle.edu.au (C.J.S.); mark.lucock@newcastle.edu.au (M.L.); emma.beckett@newcastle.edu.au (E.L.B.); 2 School of Medicine and Public Health, University of Newcastle, Ourimbah 2258, Australia; martin.veysey@hyms.ac.uk; 3 Hull York Medical School, University of Hull, Hull HU6 7RX, UK; 4 School of Biomedical Sciences and Pharmacy, University of Newcastle, Callaghan 2308, Australia; simon.keely@newcastle.edu.au; 5 Hunter Medical Research Institute, New Lambton Heights 2305, Australia

**Keywords:** BMI, bitter, epicatechin, phenol, obesity, taste genetics, taste receptors

## Abstract

Globally, more than one-third of adults are overweight. Overweight and obesity are complex and multifaceted conditions, associated with an increased risk of chronic illness and early mortality. While there are known risk factors, these alone do not fully explain the varying outcomes between individuals. Recently, taste receptors have been proposed to have a role in the risk for obesity. These receptors are expressed throughout the gastrointestinal tract. In this system, they may be involved in modulating dietary intake and metabolic processes. The taste 2 family of receptors (T2Rs) detects bitter compounds. Receptors T2R4 and T2R5 detect (-)-epicatechin (epicatechin), an antioxidant polyphenol, which may have protective effects against obesity. However, the potential role for taste receptors in this association has not been explored. This study assessed whether polymorphisms in *TAS2R4* (rs2233998 and rs2234001) and *TAS2R5* (rs2227264) were associated with body mass index (BMI). Genotyping (Taqman qPCR assays) was performed on DNA extracted from blood samples (*n* = 563) from an elderly cohort. Homozygosity for the minor allele of all polymorphisms was significantly associated with a lower BMI in males. The *TAS2R4*-rs2233998 CC genotype, the *TAS2R4*-rs2234001 CC genotype and the *TAS2R5*-rs2227264 TT genotype were associated with lower BMI (2.1, 2.1 and 2.2 units; *p* = 0.002, 0.003 and 0.001, respectively). Epicatechin intake was not associated with BMI and genotype was not associated with epicatechin intake. This suggests that the association between *TAS2R* genotype and elevated BMI risk occurs through altered extra-oral responses and not directly via altered epicatechin intake.

## 1. Introduction

Catechins are part of a large group of plant polyphenols with exceptional antioxidant properties [1]. Interestingly, these compounds may have protective properties against obesity. A catechin-rich grape seed extract has been reported to significantly reduce body weight in mice with high-fat diet-induced obesity [2], while green tea catechins have been shown to reduce BMI, body weight and waist circumference in humans [3]. For (-)-epicatechin specifically (referred to as epicatechin throughout), murine studies have shown that epicatechin administration can reverse the negative effects of maternal obesity [4]. In humans, it has been demonstrated that epicatechin administration before a meal increased satiety [5], and further that epicatechin improved post-prandial fat and carbohydrate metabolism [6]. Altogether, there is evidence to suggest that catechins, and specifically epicatechin, may be protective against obesity. 

Globally, more than 39% of adults are overweight and more than 13% are obese [7,8,9]. However, in Australia, more than 67% of adults are overweight [7]. Interestingly, obesity rates are increasing regardless of geographic location or socioeconomic status [8]. Importantly, obesity in the elderly is associated with earlier mortality relating to comorbidities such as hypertension, diabetes and heart disease [10,11,12]. Obesity is a complex and multifaceted disease that is not fully understood. However, there have been advancements in the investigations into the genetics of obesity [13], in particular the potential role of taste genetics on dietary intake and metabolism. This study explores the relationship between taste genetics, body mass index (BMI) and epicatechin intake.

Bitter taste receptors (T2Rs) are a family of receptors responsible for the detection of bitter compounds and potential toxins. Humans have 25 functional T2Rs which, when combined, are capable of detecting hundreds of bitter compounds [14,15,16]. In the oral cavity, genetic variation in these receptors influences oral detection, food preference and intake [17,18,19,20]. Importantly, these receptors are also expressed throughout the gastrointestinal tract, where they are thought to be involved in the modulation of appetite and satiation [17,21,22], gut motility [21,22,23] and glucose homeostasis [24]. In addition, functional T2R variants are associated with obesity in a porcine model [25]. Overall, bitter taste genetics may be associated with obesity via the modulation of dietary intake and/or by the regulation of gastrointestinal hormones and gut function [26]. 

*TAS2R38* is a widely studied taste gene responsible for the detection of the bitter compounds phenylthiocarbamide (PTC) and 6-n-propyl-2-thiouracil (PROP) [27]. These compounds are commonly used as tools to detect taste phenotype. Three single-nucleotide polymorphisms (SNPs) give rise to two common forms of the gene. These polymorphisms are part of a haploblock and result in the amino acid substitutions proline-alanine-valine (PAV; associated with tasting PTC and PROP) or alanine-valine-isoleucine (AVI; associated with not tasting PTC or PROP). From this, there are three genotypes associated with taste sensitivity. PAV homozygotes can detect and respond strongly to PTC and PROP and are classified as super-tasters, heterozygotes are classified as tasters and AVI homozygotes cannot detect these compounds and are classified as non-tasters. It is important to note that *TAS2R38* genotype alone does not determine the ability to taste PTC and PROP [28]. However, it is still used as a general marker of taste acuity [29]. 

The *TAS2R38* genotype associated with non-taster status has been linked to significantly higher BMIs and/or increased dietary intake [30,31,32,33,34,35,36,37]. However, some studies report no association [38,39,40] and others report inverse associations [41]. These results may also vary with age and sex [37,39,42]. For example, in a study of 381 females and 348 males, the *TAS2R38*-rs1726866 T allele (non-taster) was associated with eating disinhibition in adult women [17]. Conversely, a study in 81 children found a significant relationship between tasters and high BMI, but reported no differences in energy intake [41]. Another study in children (*n* = 53) which compared taster status to weight-for-height percentiles, found that taster females had a significantly higher weight for height compared to non-taster females and, contrastingly, that non-taster males had a higher weight for height than male tasters [42]. Furthermore, a study in 118 elderly Polish women found no significant correlation between *TAS2R38* genotype and BMI [39]. Importantly, the relationship between bitter sensitivity and BMI is known to vary with age [43]. In a cross-sectional study of 311 men and women, it was found that individuals under 65 with a higher BMI (>28) were less sensitive to bitter taste. However, in the over 65 group, overweight subjects were more sensitive to bitter taste [43]. Overall, bitter sensitivity, and the relationship between *TAS2R38* genotype and BMI may vary with age and sex.

A group of bitter receptors, T2R4, T2R5 and T2R39, detect epicatechin [44]. Therefore, we analysed three common *TAS2R* polymorphisms that result in functional receptor changes. TAS2R39 was not analysed due to very low polymorphism frequency in this gene [45]. Two common polymorphisms in the *TAS2R4* gene (rs2233998 and rs2234001) and one polymorphism in the *TAS2R5* gene (rs2227264) were assessed. These three SNPs are part of a haploblock on chromosome 7 and have previously been linked to perceived bitterness of coffee [46]. This study explores the multidirectional interactions between *TAS2R* genotype, epicatechin intake, and BMI together in an elderly cohort. 

## 2. Materials and Methods

### 2.1. Subjects and Data Collection

This study was a secondary analysis of cross-sectional data from the Retirement Health and Lifestyle Study, which was conducted on the NSW Central Coast of Australia from 2010 to 2012 [47]. Participants over the age of 65 were randomly selected from the Wyong and Gosford local areas, resulting in a cohort of primarily Caucasian heritage. This cohort was selected for this analysis to investigate the long-term effects of genotypes that correspond to functionally compromised taste receptors on BMI and dietary intake. Following screening and withdrawals, there were a total of 649 participants who gave blood samples and completed food frequency questionnaires (FFQ).

Dietary information was collected using a self-reported FFQ, adapted from the validated Commonwealth Scientific and Industrial Research Organisation Human Nutrition FFQ [48]. The FFQ contained 225 food items across all food groups with questions about frequency of consumption. For this study, this number was converted into serves per day. Participants were excluded if their FFQ was deemed invalid based on extreme energy excess or deficit (>30,000 or <3000 kJ/day) this excludes participants suspected of severely under- or overestimating daily dietary intake [49,50]. Participants who reported >11 serves per day of the same food group [51], or >4 serves of the same fruit, or same nut, per day were excluded as this is not representative of the general population [52]. Following exclusions, there were a total of 563 participants eligible for this study. 

All participants supplied written informed consent. Study approval was obtained from the University of Newcastle Human Research Ethics Committee (approval number H-2008-0431).

### 2.2. Blood Samples and BMI 

Blood samples were collected in EDTA-lined tubes by a trained phlebotomist and stored at −20 °C prior to DNA extraction. BMI was calculated from participant’s weight and height [weight (kg)/height (m^2^)]. Weight was measured to the nearest 0.01 kg using digital scales (Wedderbum© UWPM150 Platform Scale). Height was measured using the stretch stature method [53] and recorded to the nearest 0.01 cm.

### 2.3. Genotyping

Participant DNA was extracted from frozen blood samples using the QIAGEN QIAmp DNA mini kit following the manufacturer’s whole-blood protocol [54,55]. DNA samples were stored at 20 °C prior to genotyping. Genotyping was carried out via qPCR (QuantStudio 7 Flex Real-Time PCR) with TaqMan™ SNP Genotyping Assays (Applied Biosystems™, ThermoFisher Scientific, CA, USA) and TaqMan™ Genotyping Master Mix according to the TaqMan™ user guide [56,57]. Participants were included in this study only upon successful genotyping. 

### 2.4. Epicatechin Intake

Daily epicatechin intake data were estimated from a polyphenol database [58] and the FFQs [52]. The Phenol Explorer database contains the average mg/100 g of epicatechin for a large variety of foods and beverages [58]. In this study, foods that contained over 0.1 mg/100 g epicatechin from the Phenol Explorer database [58] were considered high-epicatechin foods, and were used as an indicator of epicatechin intake. This approach was used to estimate relative intake as it is notably difficult to estimate actual intake amounts (mg/day) [59]. This is primarily due to bias in self-reported dietary data and large variation in reported concentrations of epicatechin within foods [58,59] (Table 1).

The food items that fit into an FFQ category, and also contained >0.01 mg/100 g epicatechin according to the Phenol Explorer database, were included in this study (Table 1). These foods included teas, chocolates, wines, many fruits, nuts and legumes (Table 1). 

Due to the resolution of the FFQ, in this analysis, all teas, including black, green and herbal, were analysed as a group (i.e., total serves of tea per day). Additionally, all chocolate, including milk, dark, chocolate bars and chocolate biscuits were grouped together (i.e., total serves of chocolate products per day). FFQ data were available for individual wines, fruits, vegetables and nuts (i.e., serves per day of individual products). 

### 2.5. Statistics

Statistical analyses were performed using JMP (version 14.2.0, SAS Institute Inc., Cary, NC, USA). The relationship between genotype and BMI was examined using standard least squares regression. All analyses were adjusted for age and sex, or adjusted for age and stratified by sex. BMI was reported as least squares means with 95% confidence intervals. Genotypes were combined to analyse presence vs. absence of the major allele according to the TOPMED database. Dunnett’s post-hoc analysis was used to determine statistically significant differences between genotypes (*p* < 0.05). The relationship between energy intake and serves of high-epicatechin foods per day, and the relationship between BMI and serves of high-epicatechin foods per day was analysed using least squares regression. *p*-values and standardised beta values (β) were reported for correlation. Graphs were presented using Graphpad Prism (version 7.01, GraphPad Software, La Jolla, CA, USA).

## 3. Results

A total of 563 participants were included in this study following exclusions (Table 2). Of these, 254 were male and 309 were female. The average overall age was 77.4. In men, the average age was 77.4; and in women, the average age was 77.3 The average BMI for men was 28.5 and 28.6 for women, with an average of 28.5 overall. The average overall energy intake was 8223.5 kJ (8453.1–7993.9). There was a significant difference in average male daily energy intake (8656.2 kJ (8311.3–9001.1)) compared to females (7866.7 kJ (7563.1–8170.3)). The average number of serves of high-epicatechin foods per day was 5.2 for men, women and overall.

The genotype distributions are shown in Table 3. The *TAS2R4* rs2233998 polymorphism has a minor allele frequency (MAF) of 0.42. 21% of participants were homozygous for the minor allele (CC), 25% were homozygous for the major allele (TT) and 54% of participants were heterozygotes. In the rs2234001 polymorphism, 20% of participants were homozygous for the minor allele (CC; MAF = 0.48), 25% were homozygous for the major allele (GG) and 55% were heterozygotes. In the *TAS2R5* rs2227264 polymorphism, 21% of participants were homozygous for the minor allele (TT; MAF = 0.44), 27% of participants were homozygous for the major allele and 62% were heterozygotes.

**Figure 1:**
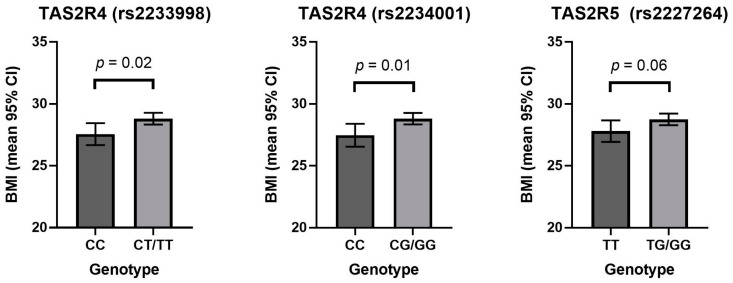
The relationship between *TAS2R* genotype and BMI. Data are presented as the mean BMI with 95% confidence intervals, adjusted for age and sex.

Homozygosity of the minor allele was associated with significantly lower BMI in both *TAS2R4* polymorphisms (Figure 1). The *TAS2R4*-rs2233998 CC genotype was associated with an average BMI of 27.7 (95% CI [26.8, 28.6]) and the presence of the G allele was associated with significantly larger average BMI of 28.7 (95% CI [28.2, 29.2]; *p* = 0.02). The *TAS2R4*-rs2234001 CC genotype was similarly associated with a lower BMI (27.6 (95% CI [26.7, 28.5])) compared to the presence of the G allele (28.8 (95% CI [28.3, 29.2]); *p* = 0.01). The presence of the major allele in the *TAS2R5* polymorphism (rs2227264) was not associated with a significant difference in BMI in this cohort.

**Figure 2:**
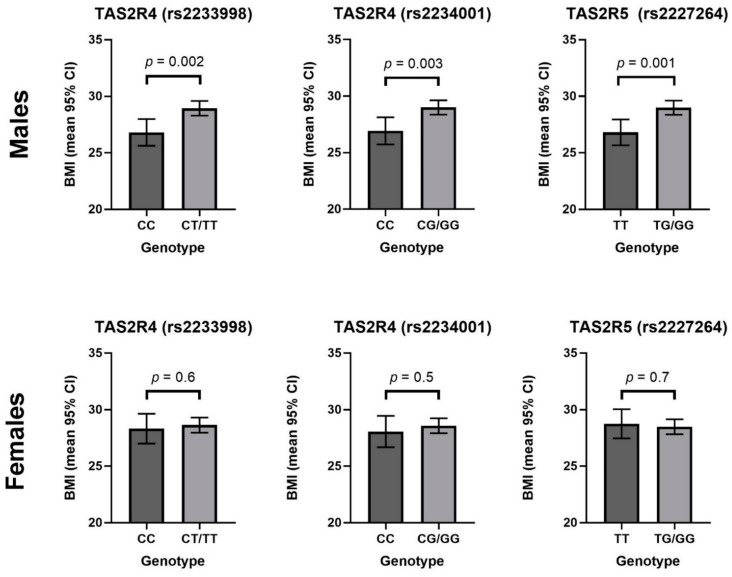
The relationship between *TAS2R* genotype and BMI by sex. Data are presented as the mean BMI with 95% confidence intervals, adjusted for age.

The relationship between BMI and TASR genotype was sex specific (Figure 2). For all three polymorphisms (rs2233998, rs2234001 and rs2227264), the presence of the major allele was associated with a significantly higher BMI than in males homozygous for the minor allele. The *TAS2R4*-rs2233998 CC genotype was associated with a BMI of 26.8 (25.6–28.0) and the presence of the G allele was associated with a significantly higher BMI (28.9 (28.3–29.6); *p* = 0.002). The *TAS2R4*-s2234001 CC genotype was associated with a BMI of 26.9 (25.7–28.1), whereas the presence of the G allele was associated with a significantly higher BMI of 29.0 (28.4–29.7; *p* = 0.003). Finally, the *TAS2R5*-rs2227264 TT genotype was associated with a BMI of 26.8 (25.7–28.0) compared to 29.0 (28.4–29.6; *p* = 0.001). In females, there was no significant difference in BMI of individuals homozygous for the minor allele, compared to the presence of the major allele. Additionally, these results remained significant when adjusted for daily energy intake.

Due to the significant difference in energy intake between males and females (Table 2), the data were analysed for a relationship between *TAS2R* genotype and daily energy intake, this analysis was stratified by sex (Table 4). The presence of the major allele was not associated with a significant different energy intake compared to homozygosity for the minor allele in any of the three polymorphisms. When stratified by sex, there was also no significant differences between the genotypes analysed.

**Figure 3:**
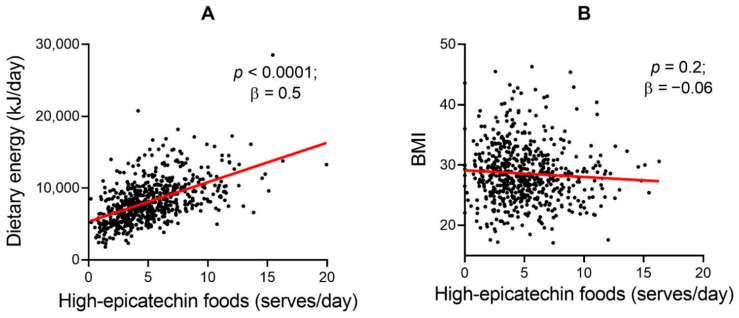
Epicatechin intake is significantly associated with dietary energy intake (**A**), but is not associated with BMI (**B**). Standardised β and p values reported for correlation.

There was a significant correlation between increased daily energy intake and increased epicatechin intake (*p* < 0.0001; β = 0.5) (Figure 3A). There was no significant relationship between BMI and serves of high-epicatechin foods per day (*p* = 0.2; β = −0.06) (Figure 3B). Additionally, there was no significant association between dietary energy intake per day and BMI (*p* = 0.5; β = −0.03) in this cohort.

There was no significant association between *TAS2R* genotype and the average number of serves of high-epicatechin foods (Table 5). However, there was consistently higher epicatechin intake observed in males homozygous for the minor allele of all three polymorphisms (compared to male carriers of the major allele and both female groups). Interestingly, these are the same groups associated with significantly lower BMIs in Figure 2.

## 4. Discussion

The secondary analysis presented here identifies potential associations between common *TAS2R4* polymorphisms and BMI. Homozygosity for the minor alleles of *TAS2R4*-rs2233998 and *TAS2R4*-rs2234001 was associated with significantly lower BMI compared to carriers of the major allele in this cohort. The three *TAS2R*SNPs analysed in this study (*TAS2R4*-rs2233998, *TAS2R4*-rs2234001 and *TAS2R5*-rs2227264) are part of a haploblock on chromosome 7 [46]. Therefore, it was expected that the SNPs assessed may be associated with the same parameter (BMI). Importantly, the association between *TAS2R4* genotypes (*TAS2R4*-rs2233998, *TAS2R4*-rs2234001) and BMI could not be explained in this cohort by daily energy intake or by daily epicatechin intake. The lack of association between energy intake and BMI suggests *TAS2R4* genotypes do not modulate food intake. Alternatively, functional *TAS2R4* polymorphisms may affect the extra-oral roles of taste receptors in energy metabolism [26]. 

Importantly, this study highlights a previously unexplored potential relationship between *TAS2R4* and *TAS2R5* genotypes and BMI. In males, homozygosity for the minor allele of all three polymorphisms corresponded to a lower BMI (>2 BMI units) in each instance, this equates to several kilograms of weight difference, depending on height. The risk for conditions associated with higher BMI such as hypertension, diabetes and cardiovascular disease increases with increased BMI [8,9,60]. For example, each one-unit increase in BMI is significantly associated with a 4% risk of ischemic stroke and a 6% increase in risk of hemorrhagic stroke [61]. Additionally, in adolescent men (*n* = 37674), risk for diabetes increases by 9.8% and risk for heart disease increases by 12% per one BMI unit increase [62]. The effects of increased BMI is particularly pronounced in the elderly where overweight and obese individuals experience earlier mortality than their normal weight counterparts [10,11,12]. Overall, this study provides insight into the genetic risk factors for obesity in the elderly.

A potential role for other extra-oral bitter receptors genotypes in predicting BMI has previously been suggested [35,41]. A Korean study (*n* = 3567) identified that the *TAS2R38*-rs10246939 TT genotype (associated phenotypically with non-tasting) was associated with a significantly higher BMI in females. However, there was no association between genotype and energy intake, suggesting another biological mechanism [35]. Additionally, a study in children (*n* = 81) which found a significant association between tasters and high BMI, found no complementary relationship between taster status and energy intake [41]. When taken together with the results presented here, a potential role for extra-oral T2Rs in predicting BMI, without modulating energy intake is suggested. 

The extra-oral roles of T2R activation on appetite and gut motility may be a potential explanatory factor for these observations. Treatment with bitter taste receptor agonists has been shown to alter satiation, food intake and gastric emptying. Intra-gastric administration of 1 μmol/kg of the bitter taste receptor agonist, denatonium benzoate, significantly increased satiation in healthy volunteers (*n* = 13) [21]. Furthermore, a study in 16 women that examined the effects of chewing and then expectorating either a bitter bar or a pleasant-tasting bar determined that gastric emptying was significantly delayed in response to the bitter-tasting bar [23]. In animal and cell models, it has been identified that intestinal taste receptors modulate the secretion of gastrointestinal hormones GLP-1, GIP, ghrelin, CCK and PYY [21,22,23,24,26,63,64] involved in appetite, digestion and glucose homeostasis [21,22,24,63,64,65]. Therefore, functional extra-oral receptor changes related to *TAS2R* genotype may influence the secretion of gastrointestinal hormones in response to bitter agonists and impact obesity risk. However, additional studies are needed to determine the causative mechanism(s). 

Although there was no association between epicatechin intake and BMI in this cohort, the administration of epicatechin (detected by T2R4 and T2R5) has previously been associated with improved cardiometabolic function [6,66]. A study in 20 adults found that following 1 mg/kg epicatechin ingestion, lipid oxidation was significantly increased in overweight subjects and post-prandial triglyceridemia decreased in normal and overweight subjects [6]. Another small study (12 males) reported significantly improved vascular function following 1–2 mg/kg body weight oral dose of epicatechin [66]. However, results from the present study suggest that nutritive doses of epicatechin did not have an effect on BMI in elderly subjects. Similarly, a previous study identified that a nutritive dose of 25 mg/day had no effect on cardiometabolic factors (blood pressure, glucose, insulin, insulin resistance, triglycerides, or total LDL, or HDL cholesterol) [67]. 

Interestingly, the number of serves of high-epicatechin foods per day was associated with increased daily energy intake in this study. It may be that higher epicatechin intake in this study is simply a function of higher overall food intake. By contrast, it has previously been demonstrated in humans that epicatechin administration before a meal increased satiety [5]. Additionally, while *TAS2R38* genotypes have previously been associated with altered oral detection, food preference and intake [17,18,19,20], there was no significant association between *TAS2R4* and *TAS2R5* genotypes and epicatechin intake in this study. This suggests that these polymorphisms are not altering oral detection and modulating intake of epicatechin containing foods. However, functional receptor changes associated with these *TAS2R*polymorphisms may alter extra-oral metabolic responses to epicatechin. 

Associations between *TAS2R38* genotypes and BMI and associated taster status and BMI have previously been reported [30,31,32,33,34,35,36,37,41]. However, this study is unique in examining the relationship between *TAS2R4* and *TAS2R5* genotypes and BMI and supports a role for *TAS2R* genotypes in predicting BMI in males. The association between homozygosity for the minor alleles of *TAS2R4*-rs2233998, *TAS2R4*-rs2234001 and *TAS2R5*-rs2227264 and lower BMI appears to be specific to males in this cohort. Sex specificity has been previously identified between *TAS2R* genotypes and a variety of outcomes, including dietary intake [17,68], BMI, [35,42] and thyroid function, which effects metabolism [69]. The sex specificity of the observed results may be explained by potential interactions between sex hormones and taste signalling, other genes located on sex chromosomes, or social determinants of food choice that are gender specific. Further studies are needed to understand these relationships. Altogether, this study provides further evidence of a potential sex dimorphism in the relationship between *TAS2R4* and *TAS2R5* genotypes and BMI in elderly subjects. 

It is important to note that the identified relationship between *TAS2R38* and BMI may also vary with age. This study found no significant association between *TAS2R4* or *TAS2R5* genotypes and BMI in elderly women, while other studies in children [32,41] and adults [30,31,33,34,35] report potential links between *TAS2R38* genotype and BMI, and a previous study in elderly women found no significant association between *TAS2R38* genotype and BMI [39]. It is well-documented that taste loss occurs during ageing [70,71]. Therefore, further studies are needed in children and adults to determine whether the relationship between *TAS2R4* and *TAS2R5* genotypes and BMI is age specific as well as sex specific. 

The use of an elderly cohort means that these results may be specific to elderly and not necessarily applicable to younger populations. However, this cohort was useful in studying the long-term effects of *TAS2R* genotypes on BMI. Another limitation of this study included estimations of energy intake and epicatechin intake. Dietary intake estimations are limited by low-accuracy and subject bias of food frequency questionnaires [72]. Additionally, it is important to note that epicatechin intake is hard to quantify due to the high variability of food composition [59] and the large variation in reported concentrations of epicatechin within foods [58]. 

Overall, we propose that *TAS2R* genotypes, resulting in functional receptor changes, may alter metabolic hormone secretion in a sex-specific manner, with downstream effects on BMI. Additional studies in larger and more diverse age groups are needed to establish this potential association between *TAS2R* genotype(s) and BMI. Importantly, if these relationships are established, they may be used to predict obesity risk, and potentially combat conditions associated with a larger BMI in the form of personalised nutrition therapies. Ultimately, this study provides initial insight into the complex relationship between taste genetics and BMI and the potential roles for extra-oral T2Rs in obesity risk. 

## Figures and Tables

**Table 1:** Foods with high epicatechin content (≥0.1 mg/100 g; Phenol Explorer).

Groups	High-Epicatechin Foods	Average mg/100 g [58]	SD	Standard Serving Size [52]	Average mg/Serve
Tea	Tea [Green], infusion	7.9	13.7	200 mL	15.9
	Tea [Black], infusion	3.9	4.3	200 mL	7.9
Chocolate	Chocolate, dark	70.3	29.5	25 g	17.7
	Chocolate, milk	14.6	4.8	25 g	3.7
Wine	Wine [Red]	3.8	3.2	100 mL	3.8
	Wine [White]	1.0	1.4	100 mL	1.0
Fruits	Apple [Dessert], raw	8.4	3.7	150 g	12.6
	Peach, peeled	8.0	4.2	150 g	12.0
	Apple [Dessert], pure juice	7.8	7.7	150 g	11.6
	Grape [Black]	5.2	5.6	150 g	7.9
	Red raspberry, raw	5.1	3.7	150 g	7.6
	Apricot, raw	3.5	4.3	150 g	5.2
	Nectarine, peeled	3.0	1.1	150 g	4.5
	Plum, fresh	2.2	2.2	150 g	3.3
	Blueberry, raw	1.1	0	150 g	1.7
	Grape [Green]	0.5	0.5	150 g	0.7
	Avocado, raw	0.4	0.2	150 g	0.6
	Kiwi	0.3	0.2	150 g	0.4
	Banana, raw	0.1	0.1	150 g	0.2
Vegetables	Broad bean seed, raw	22.5	0	75 g	16.9
	Green bean, raw	0.7	2.7	75 g	0.5
Nuts	Lentils, whole, raw	0.1	0.3	75 g	0.1
	Cashew nut, raw	0.9	0	30 g	0.3
	Pecan nut	0.8	0	30 g	0.2
	Almond	0.6	0.4	30 g	0.2
	Hazelnut, raw	0.2	0	30 g	0.1

**Table 2:** Participant characteristics reported as the mean (95% CI).

Characteristic	Male (*n* = 254)	Female (*n* = 309)	Total (*n* = 563)
Age	77.4 (76.6–78.3)	77.3 (76.6–78.2)	77.4 (76.8–78.0)
BMI	28.5 (27.9–29.1)	28.6 (28.0–29.2)	28.5 (28.1–29.0)
Daily energy intake (kJ)	* 8656.2 (8311.3–9001.1)	* 7866.7 (7563.1–8170.3)	8223.5 (8453.1–7993.9)
Serves of high-epicatechin foods per day **	5.2 (4.9–5.6)	5.2 (4.9–5.5)	5.2 (5.0–5.4)

** High-epicatechin foods are defined as having ≥0.1 mg/100 g (Phenol Explorer [58]); * significant difference in energy intake between males and females (*p* = 0.0005).

**Table 3:** *TAS2R* genotype distributions.

SNP	Genotype	*n*	%	MAF	HWE χ^2^	HWE *p*
*TAS2R4* (rs2233998)	CC	112	21%	0.42	3.8	0.05
	CT	287	54%	-		
	TT	131	25%	-		
*TAS2R4* (rs2234001)	CC	108	20%	0.48	121.8	<0.0001
	CG	302	55%	-		
	GG	135	25%	-		
*TAS2R5* (rs2227264)	TT	120	21%	0.44	0.8	0.4
	TG	291	62%	-		
	GG	151	27%	-		

SNP = single-nucleotide polymorphism; MAF = minor allele frequency; HWE = Hardy–Weinberg equilibrium.

**Table 4:** Daily energy intake is not significantly associated with *TAS2R* genotype.

SNP	*TAS2R4* (rs2233998)			*TAS2R4* (rs2234001)			*TAS2R5* (rs2227264)		
	CC	CT/TT	*p*	CC	CG/GG	*p*	TT	TG/GG	*p*
**Mean kJ/day (95% CI)**	7934.5 (7428.0–8441.0)	8278.6 (8013.4–8543.7)	0.2	7905.2 (7386.8–8423.7)	8272.3 (8011.8–8532.8)	0.2	8056.8 (7564.0–8549.6)	8304.7 (8045.3–8564.2)	0.4
**Male mean kJ/day**(95% CI)	8530.8 (7777.1–9284.6)	8644.6 (8236.8–9052.4)	0.8	8552.8 (7794.8–9310.9)	8585.0 (8189.5–8980.6)	0.9	8574.6 (7834.9–9314.3)	8686.0 (8288.6–9083.4)	0.8
**Female mean kJ/day (95% CI)**	7350.2 (6660.4–8039.9)	7902 (7554.9–8249.3)	0.1	7282.3 (6565.9–7998.7)	7943.7 (7598.9–8288.4)	0.1	7561.5 (6896.1–8226.9)	7914.7 (7573.4–8256.0)	0.4

**Table 5:** TAS2R genotype does not significantly affect the average number of serves of high-epicatechin foods per day.

SNP	*TAS2R4* (rs2233998)		*p*	*TAS2R4* (rs2234001)		*p*	*TAS2R5* (rs2227264)		*p*
	CC	CT/TT		CC	CG/GG		TT	TG/GG	
**Mean (95% CI)**	5.3 (4.8–5.7)	5.2 (4.9–5.4)	0.7	5.3 (4.8–5.8)	5.2 4.9–5.4)	0.8	5.4 (4.9–5.9)	5.2 (4.9–5.5)	0.5
**Male mean (95% CI)**	5.5 (4.8–6.3)	5.1 (4.7–5.5)	0.3	5.4 (4.7–6.1)	5.1 (4.7–5.5)	0.5	5.6 (4.8–6.3)	5.2 (4.8–5.6)	0.4
**Female mean (95% CI)**	5.0 (4.2–5.7)	5.2 (4.8–5.6)	0.6	5.1 (4.4–5.8)	5.2 (4.9–5.6)	0.7	5.2 (4.5–5.9)	5.2 (4.9–5.6)	1.0

## Data Availability

The data presented in this study are available on request from the corresponding author. The data are not publicly available due to ethical reasons.
